# The delta subunit of the GABA_A_ receptor is necessary for the GPT2-promoted breast cancer metastasis

**DOI:** 10.7150/thno.80544

**Published:** 2023-02-21

**Authors:** Na Li, Xiang Xu, Dan Liu, Jiaxin Gao, Ying Gao, Xufeng Wu, Huiming Sheng, Qun Li, Jun Mi

**Affiliations:** 1 Hongqiao International Institute of Medicine, Tongren Hospital; Basic Medical Institute; Key Laboratory of Cell Differentiation and Apoptosis of the Chinese Ministry of Education, Shanghai Jiao Tong University School of Medicine; 2 Department of Laboratory Medicine, Shanghai General Hospital Jiading Branch, Shanghai; 3 College of Basic Medical Sciences, Dalian Medical University; 4 Department of Clinic Laboratory, Tongren Hospital, Shanghai Jiao Tong University School of Medicine; 5 Department of Oncology, Shanghai East Hospital, Tongji University School of Medicine

**Keywords:** GPT2, breast cancer, GABA, GABA receptor, metastasis, calcium

## Abstract

**Objectives:** Glutamic pyruvate transaminase (GPT2) catalyzes the reversible transamination between alanine and α-ketoglutarate (α-KG) to generate pyruvate and glutamate during cellular glutamine catabolism. The glutamate could be further converted to γ-aminobutyric acid (GABA). However, the role of GPT2 in tumor metastasis remains unclear.

**Methods:** The wound healing and transwell assays were carried out to analyze breast cancer cell migration and invasion *in vitro*. Gene ontology analysis was utilized following RNA-sequencing to discover the associated molecule function. The mass spectrometry analysis following phosphoprotein enrichment was performed to discover the associated transcription factors. Most importantly, both the tail vein model and Mammary gland conditional *Gpt2^-/-^* spontaneous tumor mouse models were used to evaluate the effect of GPT2 on breast cancer metastasis *in vivo*.

**Results:** GPT2 overexpression increases the content of GABA and promotes breast cancer metastasis by activating GABA_A_ receptors. The delta subunit GABRD is necessary for the GPT2/GABA-induced breast cancer metastasis in xenograft and transgenic mouse models. *Gpt2* knockout reduces the lung metastasis of the genetic *Gpt2^-/-^* breast cancer in mice and prolongs the overall survival of tumor burden mice. Mechanistically, GPT2-induced GABA_A_ receptor activation increases Ca^2+^ influx by turning on its associated calcium channel, and the surged intracellular calcium triggers the PKC-CREB pathway activation. The activated transcription factor CREB accelerates breast cancer metastasis by upregulating metastasis-related gene expressions, such as PODXL, MMP3, and MMP9.

**Conclusion:** In summary, this study demonstrates that GPT2 promotes breast cancer metastasis through up-regulated GABA activation of GABA_A_R-PKC-CREB signaling, suggesting it is a potential target for breast cancer therapy.

## Introduction

Increased glutamine metabolism is a hallmark of cancer. Proliferating tumor cells utilize glutamine's carbon for energy production and its nitrogen for the biosynthesis of nonessential amino acids, nucleotides, and other molecules 1. For example, renal cell carcinomas and colorectal cancers are glutamine-addicted 2, 3. Glutamine is instantly converted to glutamate by glutaminase, of which activity was also correlated with a malignant phenotype 4-10.

The reversible transamination is catalyzed by a transaminase GPT between pyruvate/glutamate and alanine/α-ketoglutarate (α-KG). GPTs play essential roles in gluconeogenesis and amino acid metabolism in many tissues, including skeletal muscle, kidney, and liver 11. GPT1 locates in the cytosol; a biomarker used clinically in liver diseases. The GPT2 protein is more abundant than GPT1, especially in muscle and fat, suggesting a distinct role of GPT2 in the metabolism and homeostasis of glucose, amino acids, and fatty acids 12. Under metabolic stress, GPT2 expression is upregulated in various tumor cells, including breast carcinomas, and the viability of pancreatic cancer cells was decreased when GPT2 activity was inhibited 13-16.

In the meantime, glutamate can also be converted into γ-aminobutyric acid (GABA) by glutamate decarboxylase. GABA is a primary inhibitory neurotransmitter and activates specific GABA receptors expressed in the central nervous system and many non-neuronal peripheral tissues 17-19. GABA receptors include three distinct classes, GABA_A_, GABA_B_, and GABA_C_. GABA_C_ receptors are classified as a subtype of GABA_A_ receptors 20, and both GABA_A_ and GABA_C_ receptors are ionotropic or channel receptors 21, 22. GABA signaling plays a vital role in cell differentiation and proliferation of peripheral organs and tumorigenesis 19, 23, 24. However, its role in tumor metastasis is controversial. The GABA was shown to promote tumor cell migration by inducing extracellular metalloproteinases (MMPs) 24-29, whereas the early studies showed GABA had a strong inhibitory effect on sympathicus-driven cancers 30-32. To date, it is still unclear about the detailed mechanism of how GABA signaling regulates tumor metastasis.

GABA_A_ receptors are heteromeric complexes composed of 2α, 2β, and either one of the γ-subunit or δ, ε, θ, π, or ρ subunit, which were encoded by GABR genes GABRA (1 to 6), GABRB (1 to 3), GABRG (1 to 3), GABRD, GABRE, GABRQ, GABRP, and GABRRs, respectively 33 34. In contrast, GABA_B_ receptors are obligatory heterodimers composed of R1 and R2 subunits 35 36. GABA_A_ receptors are reported to modulate calcium influx by associating with calcium channels 37-39. Calcium signaling is essential in breast cancers since the breast is intrinsically linked to calcium production during lactation. Calmodulin is a critical calcium sensor and regulates many protein kinases/phosphatases' activities through a Ca^2+^-dependent manner 40-43, including calcium-dependent protein kinases and calmodulin-binding proteins. The distribution of these calmodulin-regulated proteins varies among tissues 44. Identifying and characterizing these calmodulin-binding/targeting proteins are essential to define the pathway by which Ca^2+^-regulated signals are transduced.

In this study, we found that GPT2 promoted breast cancer metastasis by activating the GABA_A_ receptor, and the delta subunit is necessary for this activation. The activated GABA_A_ receptor increased calcium influx, and the latter upregulated the CREB-targeted gene expression, which drives breast cancer metastasis.

## Materials and Methods

### Tissue specimens

Clinical breast cancer samples were collected from Ruijin Hospital, affiliated with Shanghai Jiao Tong University School of Medicine. The Ruijin Hospital Medical Ethical Committee approved the clinical ethics. All patients in this study had a pathological breast cancer diagnosis before surgery and signed informed consent. All experiments were performed following the local government policy and the Helsinki declaration.

### Mice

The animal studies were approved by the Animal Care and Use Committee of Shanghai Jiao Tong University School of Medicine, and conducted in accordance with the established national and institutional guidelines for the use of laboratory animals.

### Cells and reagents

Human breast cancer cell MCF-7 and mouse breast cancer cells (PY8119) were cultured in Dulbecco's modified Eagle's medium (DMEM) (Cat # L110KJ, BasalMedia, Shanghai, China); and the human breast cancer cells BT-549 were cultured in the RPMI-1640 (Cat# L210KJ, BasalMedia, Shanghai, China). MDA-MB-453 were cultured in Leibovitz's L-15 Medium (Cat# 11415064, Thermo Fisher Scientific, CA, USA). All media were supplemented with 10% FBS (fetal bovine serum, Cat# 10270-106, Gibco, NY, USA) and 50 IU of penicillin/streptomycin (Cat# S110JV, BasalMedia, Shanghai, China) in a humidified atmosphere with 5% CO2 at 37 °C.

Antibodies against GPT2 (16757-1-AP), GAD1 (10408-1-AP), PKA (55382-1-AP), CaMKII (12666-2-AP), NF-κB p65 (10745-1-AP), and IκB (15649-1-AP) were purchased from Proteintech. Antibodies against PKC (#9372), phospho-PKC (Thr410/403) (#9378), phospho-CaMKII (Thr286) (#12716), phospho-PKA (Thr197) (#4781), phospho-NF-κB p65 (Ser468) (#3039), and phospho-IκBα (Ser32) (#2859) were purchased from Cell Signaling Technology. Antibodies against CREB (ab32515) and phospho-CREB(Ser133) (ab32096) were purchased from Abcam. 3-MPA (63768) and GABA (A2129) were purchased from Sigma. BAPTA (S7534), GABA (S4700) and 666-15 (S8846) were purchased from Selleck. Picrotoxin (HY-101391) and CGP52432 (HY-103531) were purchased from MCE.

### Western blotting

Cells were washed twice with PBS and lysed on ice for 20 min in RIPA lysis buffer supplemented with protease inhibitors [1 mM PMSF, 1 mg/L aprotinin, 1 mg/L leupeptin, and 1 mg/L pepstatin] and phosphatase inhibitors [1 mM Na_3_VO_4_ and 10 mM NaF]. The protein concentration was measured using a BCA assay kit (Cat# BCA02, Dingguo, Beijing, China). Subsequently, the PAGE-separated proteins were transferred to PVDF membranes that were then separately incubated with indicated antibodies. The blots were visualized using a LAS 4000 instrument (GE Healthcare).

### Phosphorylated protein enrichment

Phosphoprotein enrichment (Cat# BB-3108, Bestbio) was performed following the manufacturer's instructions for phosphorylated protein enrichment assays as previously described. Cells were washed three times with 0.9% saline and then lysed in prechilled lysis buffer (500 μL per 5 × 10^6^ cells) at 4 °C for 40 min. Extracts were centrifuged at 14,000 × g for 15 min at 4 °C, after which all samples were adjusted to a protein concentration of 0.25-0.5 mg/mL and passed through a phosphoprotein-enriching column. Phosphorylated proteins were eluted with 400 μL elution buffer. Samples were stored at -80 °C for mass spectrometry or immediately boiled with 1 × SDS loading buffer for 10 min and then analyzed by Western blotting.

### Real-time PCR

Total RNA was isolated with TRIzol reagent (Cat# 15596026, Invitrogen, CA, USA), and cDNA was synthesized using a PrimeScript RT Reagent Kit (Cat# RR037A, Takara, Kyoto, Japan) for real-time PCR with a mixture of oligo dT and random primers after genomic DNA elimination. Real-time PCR was performed with an ABI-7500 instrument (Applied Biosystems) to measure mRNA expression using a 2 × SYBR Green qPCR Master kit (Cat# A0001, EZBioscience, MN, USA) according to the manufacturer's instructions. The relative expression levels were calculated by determining the samples' threshold cycle (Ct) values. All data were normalized to the internal control β-actin.

The primers used for the real-time PCR analysis were as follows: GPT2, forward (5′- GGAGCTAGTGACGGCATTTCTACGA-3′) and reverse (5′-CCCAGGGTTGATTATGCAGAGCA -3′); β-actin, forward (5′-GCGGGAAATCGTGCGTGACATT-3′) and reverse (5′-GATGGAGTTGAAGGTAGTTTCG-3′); MMP2, forward (5'-CAGGCTCTTCTCCTTTCACAAC-3') and reverse (5'-AAGCCACGGCTTGGTTTTCCTC-3'); MMP3, forward (5′-CTGGACTCCGACACTCTGGA-3′) and reverse (5′-CAGGAAAGGTTCTGAAGTGACC-3′); MMP-9, forward (5'-TGGGCTACGTGACCTATGACAT-3') and reverse (5-GCCCAGCCCACCTCCACTCCTC-3'); PODXL, forward (5′-TCCCAGAATGCAACCCAGAC-3′) and reverse (5′-GGTGAGTCACTGGATACACCAA-3′); and PLAT, forward (5′-AGCGAGCCAAGGTGTTTCAA-3′) and reverse (5′-CTTCCCAGCAAATCCTTCGGG-3′).

### RNA-Seq

RNA-sequencing analysis was performed by BGI accomplished. After total RNA extraction, mRNA was isolated by Oligo Magnetic Beads and cut into tiny fragments for cDNA synthesis. Following the manufacturer's instructions, libraries were generated using the NEB Next UltraTM RNA Library Prep Kit (New England Biolabs, Ipswich, MA, USA) in the Illumina system. Sequencing was conducted using the Illumina Hiseq XTEN platform.

### Gene knockout by the CRISPR/CAS9

For knock-out GABRA subunits, the gRNAs were designed and synthesized:

GABRA1-sgRNA: AGCTGAATGTCCGATGCATTTGG;

GABRA5-sgRNA: CAACAGACTTCGGCCCGGGCTGG;

GABRB1-sgRNA: CAGGGCCCCCCGTCGACGTTGGG;

GABRB2-sgRNA: GCTGCTTTCTTTTGGCGTTGGGG;

GABRB3-sgRNA: CCACTCGATTGTCAAGCGTGAGG;

GABRD-sgRNA: ACACGCCGCGGTTCCTCCGCAGG;

GABRE-sgRNA: ACAGAGGCGTTCGTCGTACCAGG;

GABRP-sgRNA: CACTCTGGATGCCCGCCTCGTGG;

GABRQ-sgRNA: GAACGGTGCGGTACGGCATCCGG;

GABRG3-sgRNA: AAGAGTCACGTCGGTGTCTTGGG.

These gene fragments were cloned into the vector of plentiCRISPRv2. Lentiviruses were generated by co-transfection into HEK293T cells with one of the above recombinant plasmids and packaging plasmids (psPAX2 and pMD2G). After 48 h of lentivirus infection, Puromycin (1 mg/mL) was added to the cells to screen for stable cells.

### Luciferase reporter assays

A traditional dual-luciferase assay consisting of NF-κB, NFAT, or CREB-binding sites reporter was used to determine the transcription factors in response to GPT2 overexpression or GABA treatment as previously described. Briefly, cells were co-transfected with luciferase reporter constructs pGMNF-κB-Luc, pGMNFAT-Luc, pGMCREB-Luc vectors, and Renilla reporter plasmid. Twenty-four hours after transfection, the luciferase activity was examined by a dual-luciferase reporter assay system (Promega). The firefly luciferase activity was normalized to the Renilla activity. Luciferase activities are presented as folds increased over the luciferase activities in unstimulated conditions.

### Cell migration and invasion assays

BD cell culture inserts (24-well insert, 8-μm pore size) were utilized following the manufacturer's instructions. In general, cells were pre-treated with the inhibitors for 24 h. Then, cells (2 × 10^4^) suspended in a 200 μL serum-free medium were seeded into the upper chamber of the inserts. The 500 μL medium of 10% FBS was added to the lower sections. The rooms were incubated at 37 °C for 24 h. After incubation for 24 h at 37 °C, cells on the upper of the membrane were scraped by a cotton swab. Then, the membrane was fixed with 4% paraformaldehyde and stained with 0.5% crystal violet solution. Five random fields per well were counted under a light microscope, and each independent experiment was repeated at least three times. The control group normalized the migration ratio. For invasion assay, cells (5 × 10^4^) were loaded into BD cell culture inserts (24-well insert, 8-μm pore size), which were coated with Matrigel (BD Biosciences, Franklin Lakes, NJ, USA).

### Scratch assay

Cells grew to a cell density of approximately 100% in six-well plates. Straight scratches were made with 200 μL pipette tips, after which the cells were washed twice with PBS and cultured in RPMI-1640. The lengths of scratches were recorded every 24 h.

### Calcium imaging

Cells overexpressing or depleted of GPT2 were incubated in Hank's balanced salt solution containing the calcium-sensitive fluorescent dye Fluo-2/AM (Cat# F1201, Invitrogen, CA, USA) for 30 min at 37 °C, and picrotoxin or CGP52432 were added at concentrations of 100 μM and 33 μM, respectively. Fluorescence intensity was measured with an Olympus confocal laser scanning microscope (DU-897D-CS0) and MetaMorph software, where excitation was performed at 340 and 380 nm, after which a comparative analysis of the two emission values was performed. Serial scanning was performed at excitation wavelengths of 340 and 380 nm at 2 s intervals, and the fluorescence intensity of each emission from each cell was detected. The fluorescence intensity changes (F340/F380) indicate the intracellular calcium concentration.

### Liquid chromatography-mass spectrometry analysis

After treatment, one million cells were collected in 1 mL of -80 °C 80% methanol. Samples were vigorously vortexed and frozen, followed by thaw on ice. The freeze and thaw were repeated three times. Then, the supernatant was collected for HPLC analysis of GABA after centrifugation at 13,000 × g for 15 min.

One million cells were cultured with a DMEM medium (1mM Gln -13C (Sigma)) of 10% dialyzed FBS to trace the flux of metabolites. After 24 h, the cell culture medium and cells were collected separately, and the content of glutamine metabolite GABA in cells or the cell culture medium was detected. The samples were lysed as described above. The supernatant was evaporated, and the resulting metabolites were resuspended for LC-MS analysis (Thermo Fisher Q Exactive). The HILIC column (150 × 2.1 mm, 3 μm particle size; Waters Inc) was eluted with 5% mobile phase A (10 mM ammonium formate and 0.1% formic acid in water) for 1 min, followed by a linear gradient to 80% mobile phase B (acetonitrile with 0.1% formic acid) over 25 min. The raw data were processed using Thermo Xcalibur 3.0 software (Thermo Fisher).

### Histochemistry and Immunohistochemistry (IHC)

For H&E staining, the sections of human and mouse tumors were deparaffinized, hydrated with deionized water, and immersed in eosin red solution for 30 min at room temperature. Slides were washed with running water for 10 min and counterstained in hematoxylin. Immunohistochemistry staining was performed as previously described 13.

For Immunohistochemistry staining, tissue sections were incubated with GPT2 antibody (1:200, Proteintech), pCREB (1:200, Abcam), pPKC (1:200, Abcam), or MMP9 (1:200, Abcam) overnight at 4 °C following de-paraffinization, and antigen retrieval. Secondary biotin-labeled IgG was then incubated with sections for 30 min at 37 °C. Finally, diaminobenzidine (DAB) was used for visualization.

Histochemistry score (H-SCORE) is based on the percentage of positive-staining area (0 = <5%, 1 = 6% - 25%, 2 = 26% - 50%, 3 = 51% - 75% and 4 = 76% - 100%) and staining intensity (negative, weak, moderate and strong, graded as 0, 1, 2 and 3, respectively). The proportion and intensity scores were added together and the percentage of positive-staining area in each field was counted as positive area/total area × 100%.

### Breast cancer metastasis models via tail vein injection

The sixth week female C57BL/6J mice were randomly divided into Ctrl, GPT2-OE, NC, and GPT2-KD groups, with six mice in each group. 1 × 10^6^ mouse breast cancer firefly luciferase-PY8119 cells suspended in 100 μL PBS were injected into the tail-vein. After three days, the mice were injected with GABA (25 mg/kg), picrotoxin (2 mg/kg), or 666-15 (10 mg/kg) by intraperitoneal injection every other day.

Four weeks later, live animal imaging was performed. The mice were anesthetized with isoflurane (#R510-22, WRD). Each mouse was injected intraperitoneally with D-luciferin potassium salt (#ST196, Beyotime) dissolved in PBS at 150 mg/kg concentration. Ten minutes later, the mice were placed on the IVIS stage and imaged in the bioluminescence to evaluate breast cancer metastasis capacity by using an IVIS Spectrum *In Vivo* Imaging System (PerkinElmer). Living image software was used to analyze bioluminescent images.

### Mammary gland conditional* Gpt2^-/-^* tumor metastasis mouse models

To study the GPT2 effect on breast cancer metastasis *in vivo*, conditional *Gpt2* gene knockout mice in the mammary gland were generated. First, homozygous floxed *Gpt2* alleles (*Gpt2*^fl/fl^) mice were generated by means of the CRISPR/Cas9 and Cre-loxP technology. The *Gpt2* gene ID of mice was 108682 and the ATGGCGGTGAACACTAAGGTGGG and GTGCTAGGCAGGCGGGATATAGG were selected for construction of* Gpt2* guide RNA. Then, *Gpt2*^fl/fl^ mice were crossed with MMTV-PyMT transgenic mice 45, which express the polyoma virus middle T oncogene (PyMT) under the control of the mouse mammary tumor virus (MMTV) LTR promoter and serve as metastatic model of autochthonous breast cancer. All mice have been maintained on a C57BL/6 background.

PCR verified the genetic type of mice. Genomic DNA was prepared from the mouse tail using the fast tissue-to-PCR kit (#K1091, Fermentas). The primer pairs used were as follows: The *Gpt2* flox: Forward, 5′-GGATGGATGAGCCCAAATA-3′ and Reverse, 5′-AGGCTGCCACATCTTCACG-3′. DNA band was visualized on 2% agar gels stained with GelRed (41003, Biotium). All tumor and lung tissues used in this study are from female mice. For statistical analysis of mice with lung metastases from primary breast cancer, *Gpt2*^+/+^ and *Gpt2*^-/-^ MMTV-PyMT C57BL/6J mice (n = 10) at 20 weeks were sacrificed to observe the foci of lung by gross appearance and H&E staining. Tumor growth was monitored by palpation, twice per week from 8 weeks. The real death of mice or the tumor size more than 2 cm^3^ was identified as a survival endpoint to draw a Kaplan-Meier survival curve.

### Statistical analysis

The data are presented as the means ± SD. The unpaired two-tailed *t*-test and two-way ANOVA were used as indicated. Statistical significance was defined as *p* < 0.05 unless otherwise stated. Each experiment was repeated independently with similar results. *P < 0.05; **P < 0.01; ***P < 0.001.

## Results

### GPT2 promotes breast cancer metastasis

**Figure 1:**
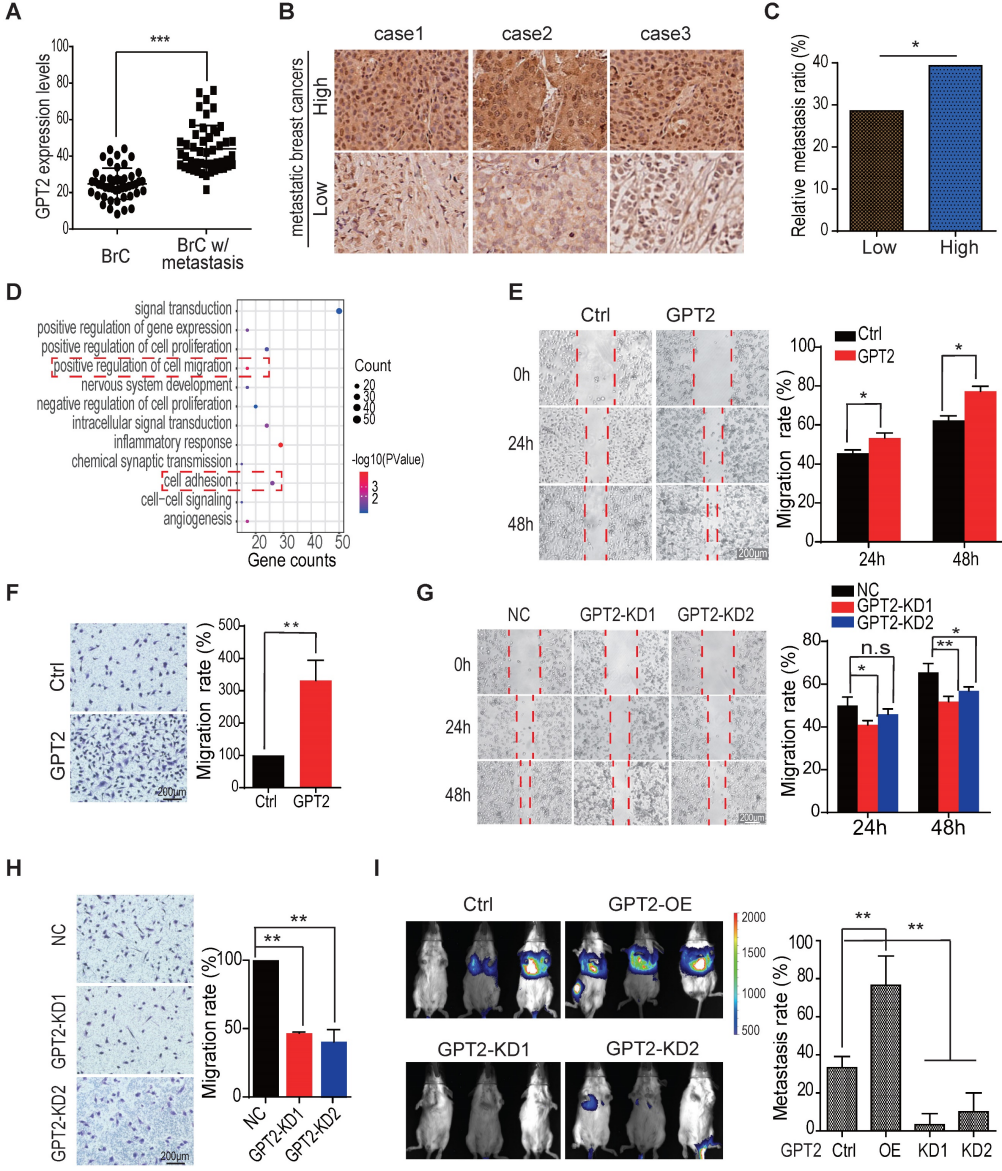
** GPT2 promotes breast cancer metastasis.** A. The GPT2 expression analysis in primary and metastatic breast cancers (n = 140), according to IHC score. “BrC” stood for primary breast cancers without metastasis; “BrC w/Metastasis” stood for breast cancer with metastasis. B. Representative immunohistochemical images of GPT2 expression in breast cancers with or without metastasis. C. According to IHC score, Breast cancer patients were divided into GPT2 low expression group (28 patients) and GPT2 high expression group (28 patients). The metastasis ratio of breast cancers with high or low expression of GPT2. D. The gene ontology analysis on the upregulated/downregulated genes determined by RNA-sequencing. The gene expression changed more than two folds were analyzed between control vs. GPT2 overexpression BT549. E-F. The effect of GPT2 overexpression on cell migration was detected by the wound healing and transwell assays. GPT2 was overexpressed in BT549 cells. G-H. The effect of GPT2 knockdown on cell migration was evaluated by the wound healing and transwell assays. GPT2 was knocked down in BT549 cells. I. The effect of GPT2 overexpression or knockdown on breast cancer metastasis in mice. Tumors were visualized by luciferase living imaging. 2 × 10^6^ Control, GPT2-overexpression and GPT2-knockdown stable Luciferase-BT549 cells were injected intravenously into female NOD/ SCID mice through the tail vain (n = 6 per group). The *in vivo* imaging was performed four weeks after injection. The graphic on the right showed the quantification of bioluminescent photon intensity.

We previously found that GPT2 promotes tumorigenesis in breast cancer 13. Then, to further investigate the association of GPT2 with breast cancer metastasis, we first analyzed the GPT2 expression by IHC in breast cancer with or without metastasis. As shown in Figure 1A, GPT2 expression was increased in metastatic breast cancers compared to primary breast cancers (p < 0.001). Figures 1B & S1A were the presentative photos of GPT2 immunohistochemistry staining. Further analysis showed that the GPT2 high expression breast cancers were more aggressive, and 40% of breast cancers with GPT2 high expression were prone to metastasis. In comparison, only 27% of GPT2 low expression breast cancers were flat to metastasis (Figures 1C & S1B), indicating that GPT2 is associated with breast cancer metastasis.

To determine whether GPT2 promotes breast cancer metastasis, we performed RNA sequencing on breast cancer BT549 cells. And the gene expression profiles were analyzed by gene ontology analysis in breast cancer cells with or without GPT2 overexpression. As shown in Figure 1D, cell migration and adhesion pathways were two of the top ten activated pathways, suggesting that GPT2 was involved in the metastatic process of breast cancer. Furthermore, the wound healing assay, transwell evaluation, and invasion test showed that GPT2 overexpression significantly promoted migration and invasion of breast cancer BT549 cells (Figures 1E, 1F & S1C); in contrast, depletion of GPT2 inhibited migration and invasion of breast cancer BT549 cells (Figures 1G, 1H & S1D).

In the meantime, we found that GPT2 overexpression also accelerated the migration of breast cancer MCF7 cells (Figure S1E). In contrast, GPT2 depletion reduced the migration of breast cancer MDA-MB-468 cells (Figure S1F), representing a high endogenous GPT2. Moreover, BT549 cells overexpressing GPT2 were more efficient in metastasis after a tail vein injection (Figure 1I). These observations suggested that GPT2 may promote breast cancer metastasis.

### GABA mediates GPT2-promoted migration and invasion of breast cancer cells

**Figure 2:**
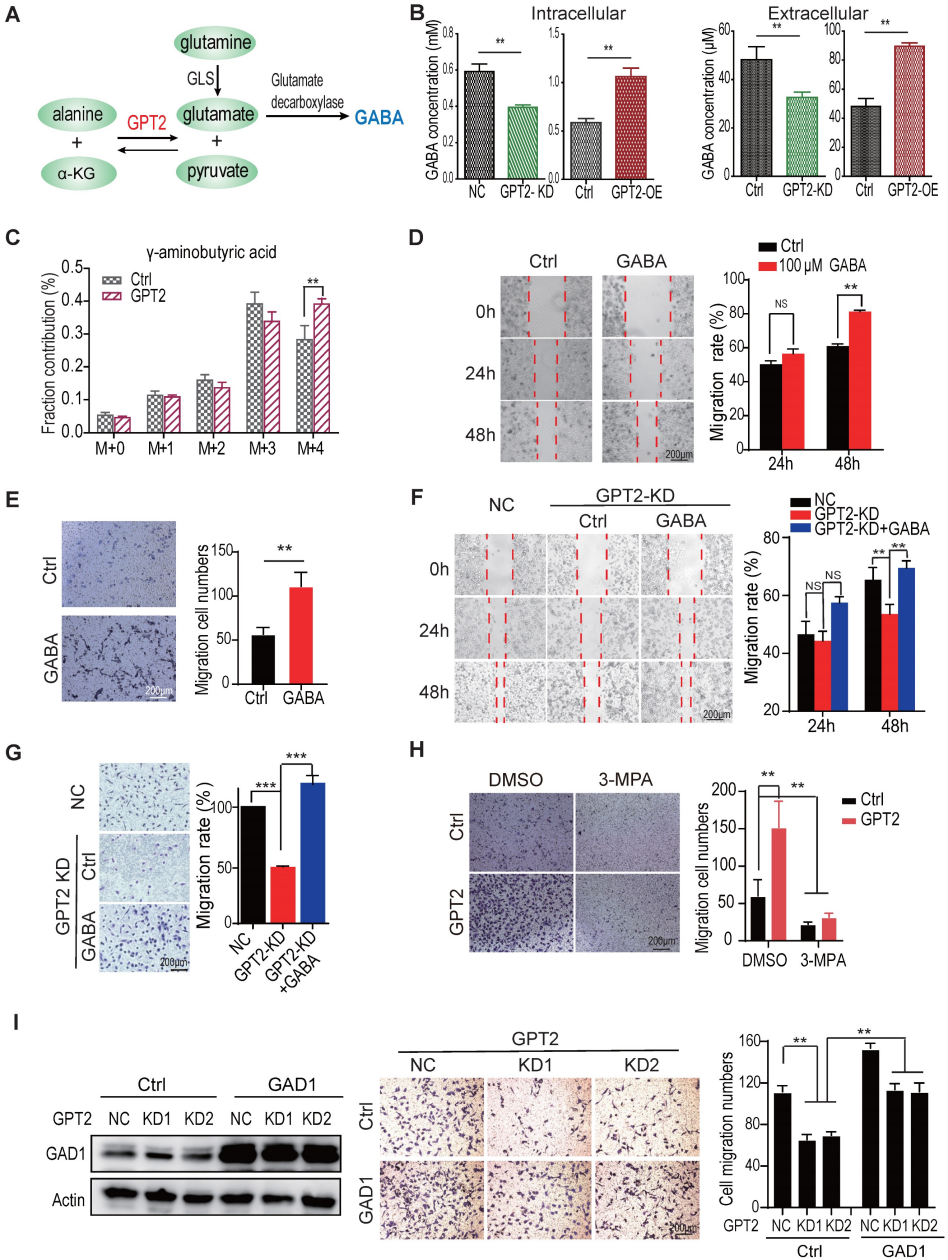
** GABA mediates GPT2-promoted migration and invasion of breast cancer cells.** A. The schematic representation of intracellular glutamine metabolic flux in cells. B. Effects of GPT2 overexpression or knockdown on intracellular or extracellular GABA content in BT549 cells, analyzed by LC-MS. C. GPT2 overexpression enhanced the metabolic influx of ^13^C-glutamine to GABA analyzed by LC-MS. D-E. The effect of GABA on cell migration of BT549 was assessed by the wound healing and transwell assays. For the transwell assay, cells were pretreated with 100 μM of GABA for 24 h. F-G. GABA rescued the GPT2 knockdown-inhibited cell migration. BT549 cells were pretreated for the transwell assay with 100 μM of GABA for 24 h. H. The GAD1 inhibitor 3-mercaptopropionic acid (3-MPA) blocked GPT2 overexpression-enhanced cell migration, detected by transwell assay. BT549 cells were pretreated with 100 μM of 3-MPA for 24 h. I. GAD1 overexpression rescued the GPT2 knockdown-inhibited cell migration in BT549 cells.

As a schematic drawing in Figure 2A, GPT2 is a critical enzyme participating in glutamine metabolism; thus, the glutamine metabolites were analyzed by LC-MS/MS to determine the mechanism GPT2 promotes breast cancer metastasis. As shown in Figure 2B, the intercellular and extracellular GABA concentration decreased in BT549 cells depleted GPT2. In contrast, the GABA content increased in BT549 cells expressing GPT2. Moreover, the glutamine metabolic flux analysis confirmed that GPT2 overexpression increased intracellular GABA production, suggesting GPT2 increased GABA content by increasing glutamate concentration (Figure 2C). Since GABA is an important signal molecule, we proposed that GABA might regulate breast cancer metastasis.

To determine whether GPT2 promoting breast cancer cell migration depends on GABA, we assessed the migration and invasion capability by the wound healing evaluation and the transwell assay in BT549 cells treated with GABA. As shown in Figures 2D-E & S2A, GABA promoted breast cancer cell migration and invasion. Moreover, GABA recovered the migration and invasion ability of breast cancer cells depleted of GPT2 (Figures 2F, 2G & S2B). Moreover, the conditional media from GPT2 overexpressing BT549 cells significantly promoted the cell migration of breast cancer cells depleted of GPT2 compared to the media from parental cells (Figure S2C).

Most importantly, the GABA catabolic enzyme GAD (glutamate decarboxylase) inhibitor 3-mercaplopropionic acid (3-MPA) significantly suppressed breast cancer cell migration (Figure 2H). In the meantime, GAD1 overexpression facilitated breast cancer migration. And the GAD1 overexpression also partially recovered the GPT2 depletion-reduced breast cancer migration (Figure 2I). These observations suggested that GABA as a signal molecule mediates GPT2 promotion of breast cancer cell migration.

### The delta subunit is necessary for GABA_A_ receptor-mediated breast cancer migration

**Figure 3:**
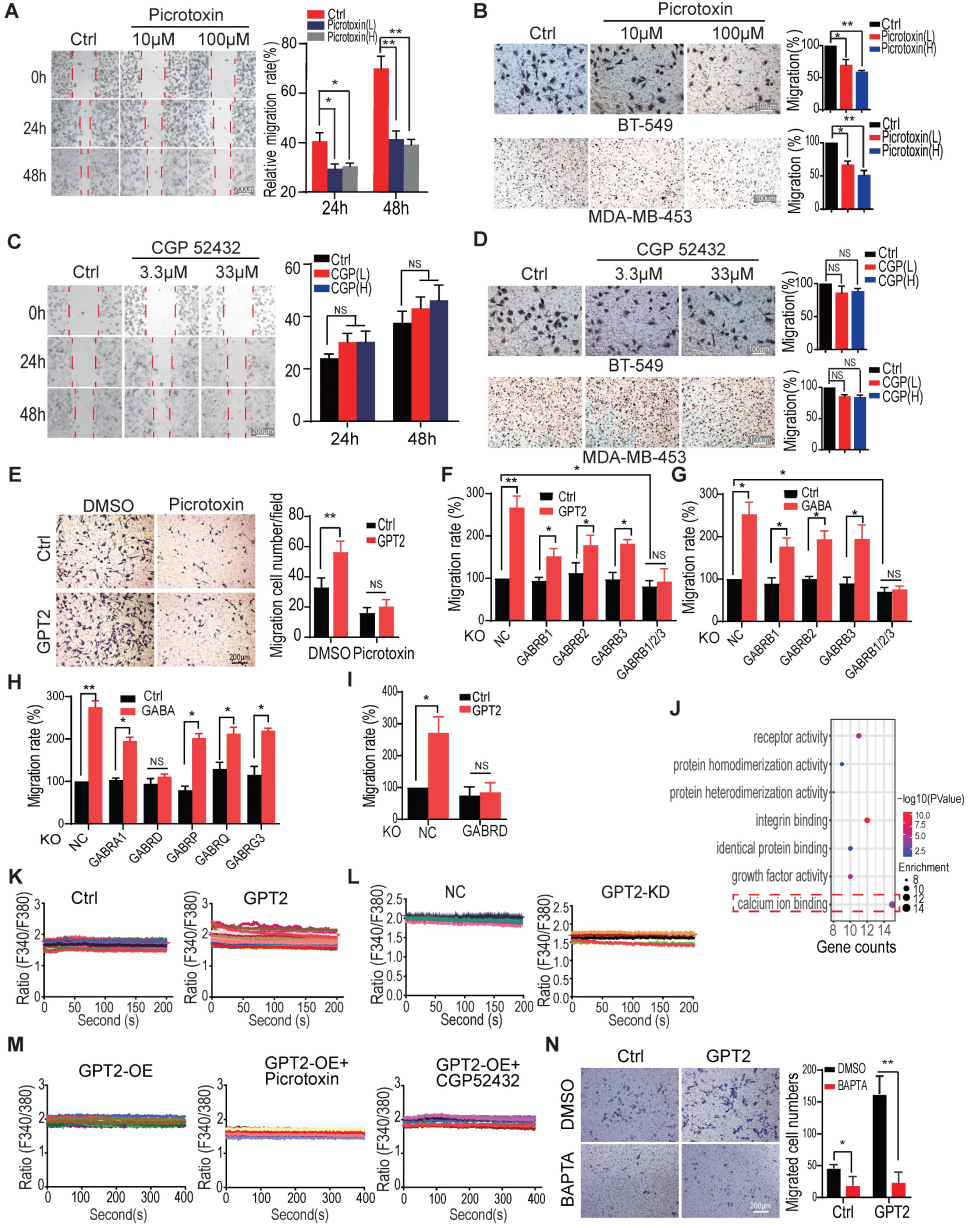
** The delta subunit is necessary for GABA_A_ receptor-mediated breast cancer migration.** A. The GABA_A_ receptor inhibitor picrotoxin decreased BT549 cell migration, assessed by the wound healing assays. The concentration of GABA_A_ receptor inhibitor Picrotoxin were 10 μM and 100 μM, respectively. B. The GABA_A_ receptor inhibitor picrotoxin decreased BT549 and MDA-MB-453 cell migration, evaluated by transwell assay. Cells were pretreated with 10 μM and 100 μM picrotoxin for 24 h. C. The GABA_B_ receptor inhibitor CGP 52432 did not affect BT-549 cell migration, as determined by the wound healing assays. The concentration of GABA_B_ receptor inhibitor CGP 52432 were 3.3 μM and 33 μM, respectively. D. The GABA_B_ receptor inhibitor CGP 52432 did not affect BT549 and MDA-MB-453 cell migration, tested by transwell assay. Cells were pretreated with 3.3 μM and 33 μM CGP 52432 for 24 h. E. The GABA_A_ receptor inhibitor picrotoxin blocked GPT2-enhanced cell migration in BT549, evaluated by transwell assay. Cells were pretreated with 100 μM of picrotoxin for 24 h. F-G. Knockout of GABRB1, GABRB2, and GABRB3 simultaneously abolished GABA (F) and GPT2 (G) -induced breast cancer cell migration in BT549, analyzed by transwell assay. Cells were pretreated with 100 μM GABA for 24 h in F. H. The effect of GABA on cell migration was detected by transwell assay in BT549 cells depleted of GABRA1, GABRD, GABRP, GABRQ, or GABRG3. I. The knockout of GABRD abolished GPT2-induced breast cancer cell migration in BT549, which was detected by transwell assay. J. The gene ontology analysis on the upregulated/downregulated genes by GABA treatment. The RNA-sequencing was performed, and the gene expression changed more than two folds were analyzed. BT549 cells were treated with 100 μM of GABA. K. GPT2 overexpression enhanced cellular Ca^2+^ concentration in BT549. The calcium concentration was measured by calcium imaging assay using calcium-sensitive dye Fluo-2/AM. L. GPT2 knockdown decreased cellular Ca^2+^ concentration in BT549 cells. M. Picrotoxin, but not CGP 52432, blocked GPT2-enhanced cellular Ca^2+^ concentration in BT549. Cells were pretreated with 100 μM of picrotoxin or 33 μM of CGP 52432 for 24 h. N. Calcium chelator BAPTA blocked GPT2-enhanced cell migration in BT549. Cells were pretreated with 1 μM of BAPTA for 24 h.

To determine whether the GABA-induced cell migration/metastasis depends on the GABA receptors, we tested the cell migration capability by the wound healing evaluation and transwell assay in breast cancer cells expressing GABA receptors. These cells were separately treated with the GABA_A_ receptor inhibitor picrotoxin or the GABA_B_ receptor inhibitor CGP 52432. As shown in Figures 3A-B, the GABA_A_ receptor inhibitor picrotoxin significantly suppressed GABA-induced cell migration in two breast cancer cells in a dose-dependent manner (p < 0.01), but not the GABA_B_ receptor inhibitor CGP 52432 (Figures 3C-D). Picrotoxin also inhibited GPT2-induced breast cancer cell migration (Figure 3E).

To further confirm that the GABA-induced cell migration depends on the GABA_A_ receptors, but not GABA_B_, we knocked out the GABA_A_-unique β subunits. The GABRB1, GABRB2, and GABRB3 were individually or entirely knocked out. We found that the individual depletion of GABRB1, GABRB2, and GABRB3, all of them suppressed GABA or GPT2-induced breast cancer cell migration, to some extent (Figures 3F/S3A-B & 3G/S3C-D). The total depletion of three subunits dramatically inhibited GABA or GPT2-induced breast cancer cell migration (Figures 3F/S3A-B & 3G/S3C-D), suggesting the GABA-induced cell migration depends on the GABA_A_ receptors, but not GABA_B_.

To explore the critical subunit(s) of the GABA_A_ receptors involved in GABA-induced breast cancer cell migration, we first analyzed the expression of GABA_A_ receptor subunits based on the TCGA database. We found that the seven GABA_A_ receptor subunits were upregulated in TNBC breast cancers, including GABRA1, GABRA5, GABRD, GABRE, GABRP, GABRQ, and GABRG3 (Figure S3E). Then, these seven upregulated GABR genes were knocked out by CRISPR/Cas9 technology to determine whether these subunits promote cell migration in TNBC cells (Figure S3F). As shown in Figure S3G, the cell migration was reduced or unchanged in BT549 cells knocked out of GABRA1, GABRD, GABRP, GABRQ, or GABRG3, but not the GABRA5 and GABRE depletion.

Only the GABRD knockout inhibited the GABA-induced cell migration in response to GABA treatment. While the knockout of GABRA1, GABRD, GABRP, GABRQ, and GABRG3 have little effect on cell migration (Figures 3H & S3H). Moreover, GABRD depletion inhibited GPT2-induced breast cancer cell migration (Figures 3I & S3I-J). Meanwhile, higher GABRD expression in lymph node positive metastases of breast cancer patients was significantly correlated with poor prognosis (Figure S3K). These results suggested that the GABA_A_ receptor mediates GPT2/GABA-induced breast cancer cell migration, and the δ subunit is necessary for this induced cell migration.

The gene ontology (molecule function) analysis showed that calcium-binding signaling was significantly activated in breast cancer cells treated with 100 µM of GABA (Figure 3J), consistent with the previous finding that GABA_A_ receptor activation couples with calcium influx 21, 22, 37-39. Thus, the intracellular calcium influx/concentrations were analyzed to determine whether calcium signaling mediates GPT2/GABA-regulated breast cancer cell migration. As shown in Figures 3K-L, GPT2 overexpression increased calcium influx/concentration while GPT2 depletion reduced calcium influx/concentration. The GABA_A_ receptor inhibitor picrotoxin but not GABA_B_ inhibitor CGP 52432 suppressed calcium influx/concentration (Figure 3M). In addition, the calcium chelator BAPTA also significantly inhibited GPT2-induced breast cancer cell migration (Figure 3N).

To exclude redox's effect on glutamine metabolism on breast cancer cell migration, we analyzed the cellular ROS (reactive oxygen species) by flow cytometry in BT549 cells overexpressing GPT2. As shown in Figure S3L, GPT2 overexpression did not significantly change cellular ROS levels. Also, the oxidative reductase NAC did not markedly affect breast cancer cell migration (Figure S3M). These observations suggested that GABA_A_ receptors mediate GPT2/GABA-induced breast cancer cell migration via modulating calcium influx.

### CREB activation is critical for GPT2/GABA-induced cell migration

**Figure 4:**
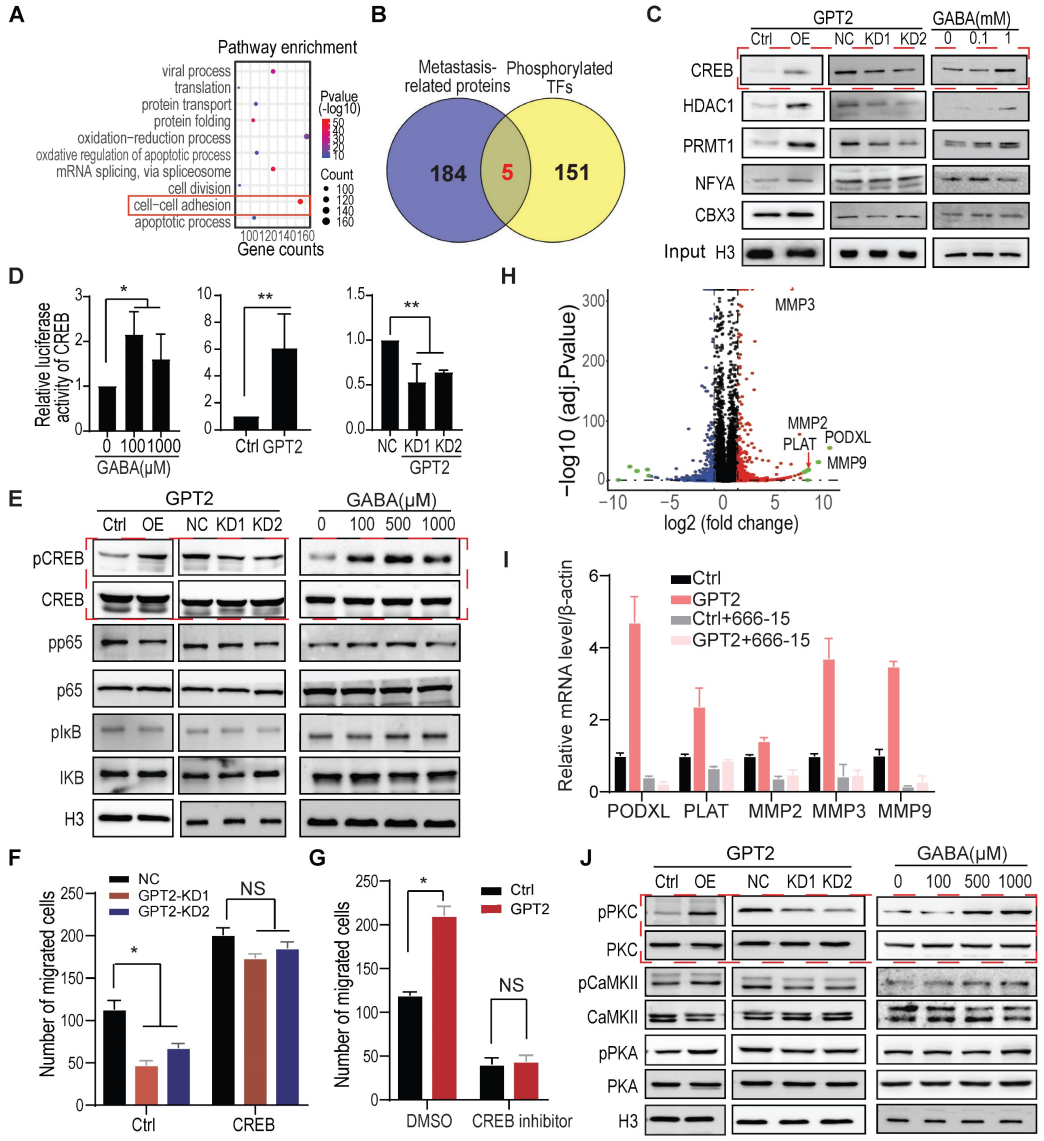
** CREB activation is critical for GPT2/GABA-induced cell migration.** A. The gene ontology analysis on the upregulated/downregulated phosphoproteins determined by mass spectrometry. The enriched phosphoproteins changed more than two folds were analyzed between control vs. GPT2-overexpressed BT549. B. The overlap of metastasis promoting proteins with phosphorylated TFs from phosphorylation enrichment analysis. The metastasis-related proteins were selected from an online database (http://hcmdb.i-sanger.com/). C. The candidate transcription factors were verified by immunoblotting in BT549 cells overexpressing or depleting GPT2 or treating GABA. The samples were collected from phosphoprotein enrichment experiments. D. Effects of GPT2 or GABA treatment on the transcriptional activity of CREB (cAMP-responsive element-binding protein) were detected in BT549. A traditional dual-luciferase assay consisting of CREB-binding sites reporter was used to detect the transcriptional activity of CREB. E. Effects of GPT2 or GABA treatment on CREB and NF-κB signaling by Western blotting in BT549. F. CREB overexpression reversed the GPT2 knockdown-inhibited cell migration in BT549. G. The CREB inhibitor 666-15 blocked GPT2-enhanced cell migration. BT549 cells were pretreated with 10 μM of 666-15 for 24 h. H. The volcano plot displayed the most upregulated/downregulated genes by GPT2. The gene expression profile was analyzed between control vs. GPT2-overexpressed BT549. I. The top 5 GPT2 upregulated genes was verified by Q-PCR analysis, and the CREB inhibitor 666-15 blocked the GPT2 upregulated genes. Cells were treated with 10 μM of 666-15 for 24 h. J. The potential kinases for CREB were verified by immunoblotting.

As a signal molecule, calcium regulates cellular function mainly through calmodulin to activate various protein kinases and protein phosphatases and consequently phosphorylate/dephosphorylate the downstream targets 44, 46, 47. Thus, the phosphorylated proteins were analyzed by protein mass spectrometry following enrichment. The gene ontology analysis of these phosphoproteins showed that the cell-cell adhesion pathway was markedly regulated after GPT2 overexpression in BT549 cells, confirming that GPT2 expression promotes tumor metastasis (Figure 4A).

Five transcription factors that potentially regulate metastasis were selected from the 156 phospho-proteins (Figure 4B). The five transcription factors, including CREB, were further verified by immunoblotting in protein samples collected from the phosphorylation protein enrichment experiments. As shown in Figure 4C, GPT2 overexpression and GABA treatment increased the phosphorylation level of one transcription factor and two histone modification enzymes among five. At the same time, GPT2 depletion decreased the phosphorylation levels (Figure 4C). Therefore, as a well-known transcription factor regulated by calcium signaling, CREB was chosen to determine whether GPT2/GABA promoted breast cancer metastasis via CREB activation.

The promoter-luciferase assay showed that GPT2 overexpression and GABA treatment significantly increased the CREB activity, but not the NF-κB nor the NFAT activity in BT549 cells. Moreover, GPT2 depletion only reduced the CREB activity (Figures 4D & S4A), which was further supported by the markers in their pathways (Figure 4E) and was consistent with the immunoblotting data (Figure 4C). Function analysis showed that CREB overexpression increased the GPT2 depletion-reduced breast cancer cell migration (Figures 4F & S4B). In contrast, the CREB inhibitor 666-15 decreased the GPT2-promoted breast cancer cell migration (Figures 4G & S4C).

The volcano graphic displayed the differentially expressed genes in BT549 cells overexpressing GPT2, including PLAT, PODXL, and MMPs (Figure 4H). The quantitative PCR showed that the GPT2-induced PODXL, MMP3 and MMP9 expression was suppressed by the CREB inhibitor 666-15 (Figure 4I). The upregulation of PODXL, MMP3 and MMP9 are believed to promote cell migration 48-50, suggesting CREB activation is critical for GPT2/GABA-induced breast cancer migration. In the end, we identified the calcium-regulated kinases that potentially phosphorylate CREB from three kinases, CaMKII, PKA, and PKC. As shown in Figure 4J, PKC activation was increased in breast cancer cells expressing GPT2 or treated with GABA. In contrast, GPT2 depletion inhibited PKC activation, indicating that PKC may phosphorylate CREB in response to GABA-induced calcium influx.

### GPT2 knockout inhibits breast cancer metastasis in mice

**Figure 5:**
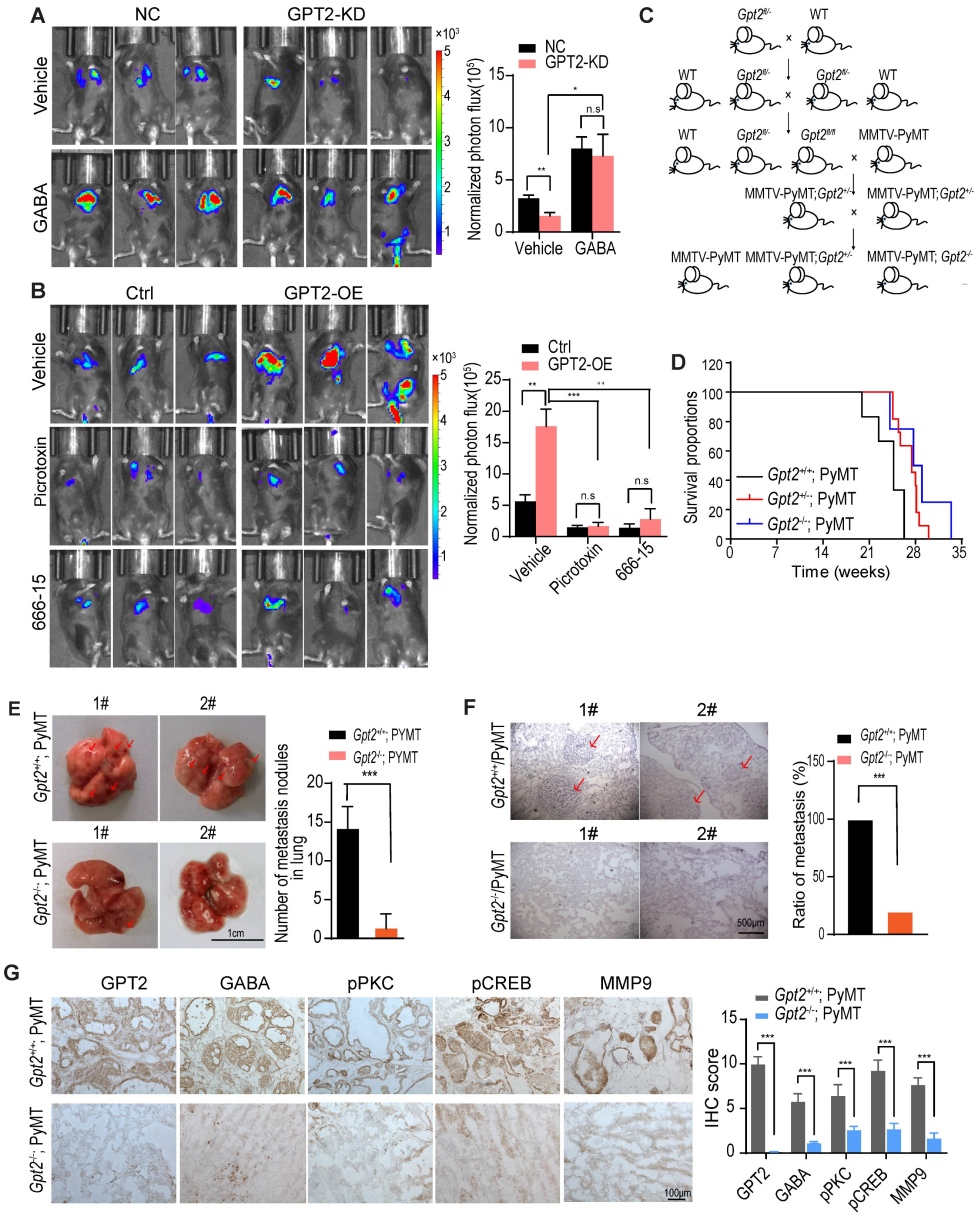
** GPT2 knockout inhibits breast cancer metastasis in mice.** A. GABA treatment reversed the GPT2 knockdown-inhibited breast cancer metastasis. The negative control or GPT2 knockdown mouse breast cancer firefly luciferase-PY8119 cells were injected into the tail-vein of C57BL/6J mice (n = 6 per group). The GABA was administered at 25 mg/kg/2 day via intraperitoneal injection. The *in vivo* bioluminescent imaging was performed four weeks post-injection. The graphic on the right showed the quantification of bioluminescent photon intensity. B. The GABA_A_ receptor inhibitor picrotoxin and the CREB inhibitor 666-15 blocked GPT2-enhanced breast cancer metastasis. The control or GPT2 overexpression mouse breast cancer firefly luciferase-PY8119 cells were injected into the tail-vein of C57BL/6J mice (n = 6 per group). Intraperitoneally injected the picrotoxin at 2 mg/kg/2 days or the 666-15 at 10 mg/kg/2 day for 4 weeks. The *in vivo* bioluminescent imaging was performed four weeks post-injection. The graphic on the right showed the quantification of bioluminescent photon intensity. C. The schematic representation of generating mammary gland conditional *Gpt*2 gene knockout breast cancer metastasis model by* Gpt*2^fl/fl^ C57BL/6 mice mated with MMTV-PyMT mice. D. Survival curves of MMTV-PyMT;* Gpt2^+/+^* and MMTV-PyMT; *Gpt2^-/-^* C57BL/6J mice, *Gpt2* knockout prolonged the overall survival of MMTV-PyMT spontaneous breast cancer mice. E. Quantification of metastatic nodules based on gross appearance confirmed that *Gpt2* knockout reduced breast tumor metastasis formed in the lungs. Representative images lung tissues were obtained from 20-weeks MMTV-PyMT; *Gpt2^+/+^* and MMTV-PyMT; *Gpt2^-/-^* C57BL/6J mice (n = 10). Arrows indicate the lung metastatic nodules. The graphic on the right showed the statistical number of metastatic foci. F. Hematoxylin and eosin stains provide histologic confirmation that *Gpt2* knockout decreased breast tumor metastasis formed in the lungs. Representative HE stainning of lung tissues was obtained from 20-weeks MMTV-PyMT; *Gpt2^+/+^* and MMTV-PyMT; *Gpt2^-/-^* C57BL/6J mice (n = 10). Arrows indicate the lung metastatic nodules. The graphic on the right showed the statistical results of mice with lung metastases from primary breast cancer. G. Representative imagines and quantitative analysis of IHC staining of GPT2, GABA, pPKC, pCREB, and MMP9 in mammary tumors from 20-weeks MMTV-PyMT; *Gpt2^+/+^* and MMTV-PyMT;* Gpt2^-/-^* C57BL/6J mice.

Finally, we utilized the mice model to verify the promoting effect of GABA/calcium/CREB signaling on tumor metastasis. As shown in Figure 5A, the GABA treatment recovered the GPT2 depletion-suppressed breast cancer metastasis. In contrast, the GABA_A_ receptor inhibitor picrotoxin and CREB inhibitor 666-15 inhibited the GPT2-promoted breast cancer metastasis (Figure 5B).

In order to investigate the effect of GPT2 on breast cancer metastasis, we first introduced *Gpt2* gene knockout C57BL/6 mice, and then mated with MMTV-PyMT mice to generate MMTV-PyMT; *Gpt2^+/-^* and MMTV-PyMT; *Gpt2^-/-^* mice (Figure 5C). As shown in Figure 5D, the knockout of *Gpt2* to some extent extended the overall survival of tumor burden mice compared to the wildtype breast cancer mice model. In the mammary gland conditional *Gpt2^-/-^* mouse model, *Gpt2* knockout significantly decreased lung metastatic nodules and prolonged the overall survival of tumor burden mice (Figure 5E-F). The immunohistochemistry staining analysis also showed that *Gpt2* knockout markedly reduced GABA synthesis, PKC and CREB activation, and MMP9 expression in mouse breast tumors (Figure 5G).

## Discussion

Many studies demonstrated that glutamine metabolism is a hallmark of cancer. Glutamine catabolism is increased in highly proliferating cells for biosynthesis and energy production 51-53. Moreover, the binding of glutamate to its receptors activates SRC family kinases and its downstream signaling, consequently promoting cell proliferation, apoptosis resistance, migration, and invasion of various cancer cell lines 54. In this study, we found that glutamate-derived GABA increases Ca^2+^ influx through GABA_A_ receptor, and the latter activates transcription factor CREB to promote breast cancer metastasis. We further determined that the GABRD (delta subunit), but not GABRP (pi subunit), is necessary for the GPT2/GABA-induced breast cancer metastasis, which was distinct from the previous finding that patients with metastatic breast cancer expressed eight times of GABRP compared to stages II-IV patients without metastasis 55. Moreover, the other study also showed that GABA promotes pancreatic cancer growth through the GABRP 56. The importance of delta subunit is rare reported.

Calcium, as a second messenger, regulates various cellular functions by binding calmodulin. PKC/CREB signaling was activated in response to GPT2-induced calcium influx and consequently promoted breast cancer metastasis. However, the selectivity of calcium influx-activated signaling is still unclear, which will be determined in the future.

Although the current data strongly support that GPT2 promotes tumor metastasis through the GABA-increased calcium influx, we could not entirely exclude the other possibility for GPT2-induced tumor metastasis since GPT2 regulates the α-KG generation. The latter is also involved in tumor metastasis 57, 58. In addition, it's well known that MDA-MB-231 cells are prone to metastasis. However, none of the GABA_A_ receptors were upregulated in these cells, suggesting there are multiple mechanisms promoting breast cancer metastasis besides GABA-triggered calcium influx.

In brief, this study demonstrated that GPT2 activated GABA_A_ receptors by increasing GABA secretion. The calcium influx-triggered CREB is critical for breast cancer metastasis, suggesting that glutamine metabolism regulates breast cancer metastasis and that the GABA_A_ receptor is a potential target for breast cancer therapy.

## Supplementary Material

Supplementary figures.
