# Supplementary material for: The delta subunit of the GABAA receptor is necessary for the GPT2-promoted breast cancer metastasis
Source: Theranostics. 2023 Feb 21;13(4):1355–69. doi: 10.7150/thno.80544 (PMC10008743; doi:10.7150/thno.80544)
Supplement: Supplementary file 1 — Supplementary figures. [file thnov13p1355s1.pdf]

Figure S1

A

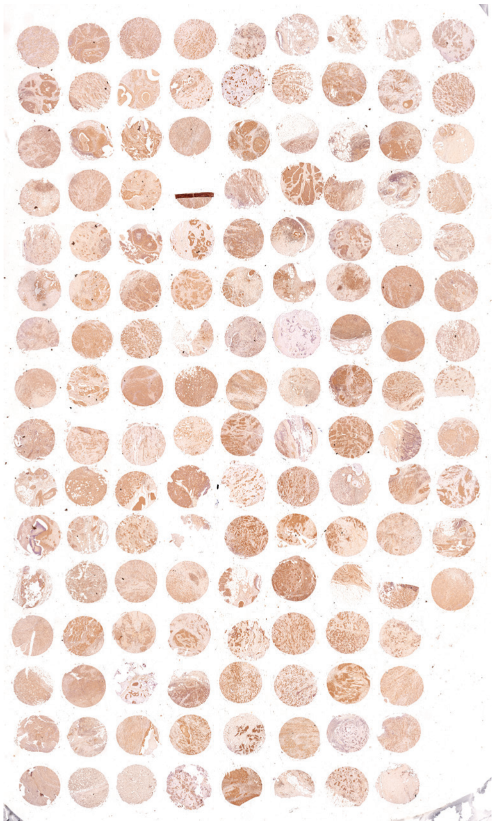

B

GPT2 expression (low)			GPT2 expression (high)		
Clinical stage	Positive lymph nodes	Colorimet density	Clinical stage	Positive lymph nodes	Colorimet density
2A	1	192	2B	2	243
2B	0	189	2A	0	239
2A	0	188	2B	2	239
2A	0	188	2B	2	236
2A	1	186	2A	0	236
2B	1	185	2B	2	235
2B	2	185	2A	2	233
2A	0	182	2A	0	232
2A	0	181	2B	1	230
2A	0	179	2A	0	228
2A	0	177	2A	0	223
2A	0	176	2A	0	223
2A	0	175	2B	1	222
2A	1	173	2A	0	222
2A	0	167	2A	0	222
2A	0	163	2A	0	220
2A	0	161	2A	2	220
2A	2	154	2A	0	219
2A	0	145	2A	0	216
2B	1	145	2A	1	213
2A	0	143	2B	2	211
2A	0	137	2A	2	211
2A	0	137	2A	0	209
2A	0	114	2A	0	206
2A	0	110	2A	0	202
2A	0	89	2A	0	202
2B	2	83	2A	0	202
2A	0	64	2A	0	201

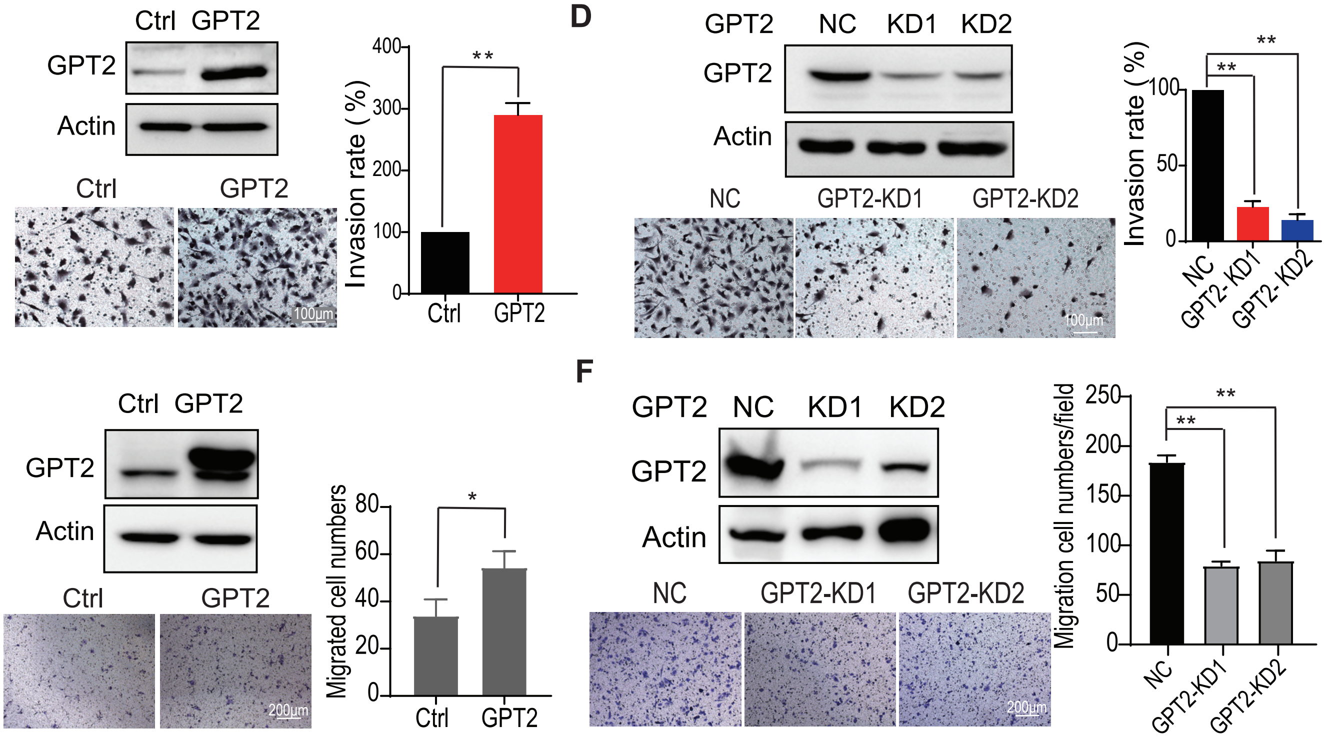

Figure S1 legend:

A. The immunohistochemistry staining of GPT2 on the tissue array of breast cancer patients.

B. The detailed metastasis information in the groups of low GPT2 expression and high GPT2 expression.

C. GPT2 overexpression promoted BT549 cell invasion. **: $p < 0.01$.

D. GPT2 knockdown suppressed BT549 cell invasion. **: $p < 0.01$.

E. GPT2 overexpression promoted MCF7 cell migration. *: $p < 0.05$.

F. GPT2 knockdwn suppressed MBA-MB-468 cell migration. **: $p < 0.01$.

Figure S2

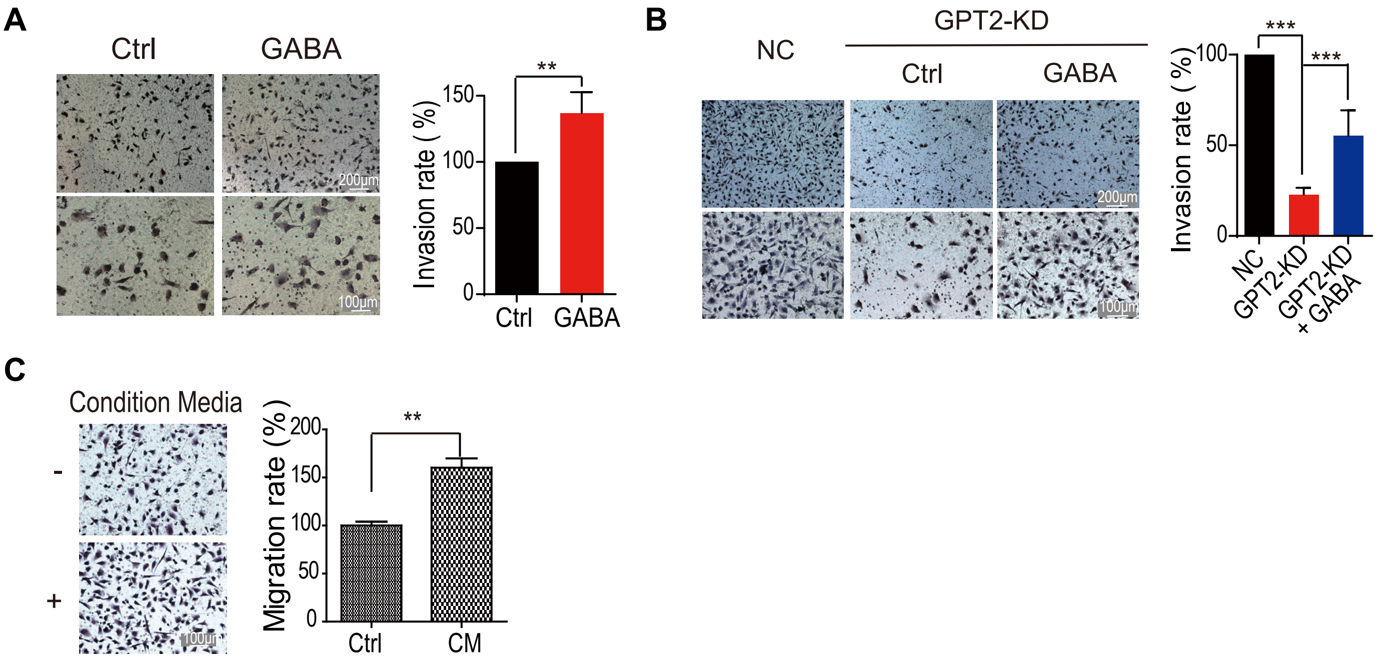

Figure S2 legend:

A. The effect of GABA on BT549 cell invasion. The BT549 cells was treated with 100 μM of GABA.

B. The effect of GABA on the cell invasion of BT549 cells depleted of GPT2. The BT549 cells were treated with 100 μM of GABA. ***: $p < 0.001$.

C. The effect of the conditional media from parental BT549 cells on the cell migration of BT549 cells depleted of GPT2. **: $p < 0.01$.

Figure S3

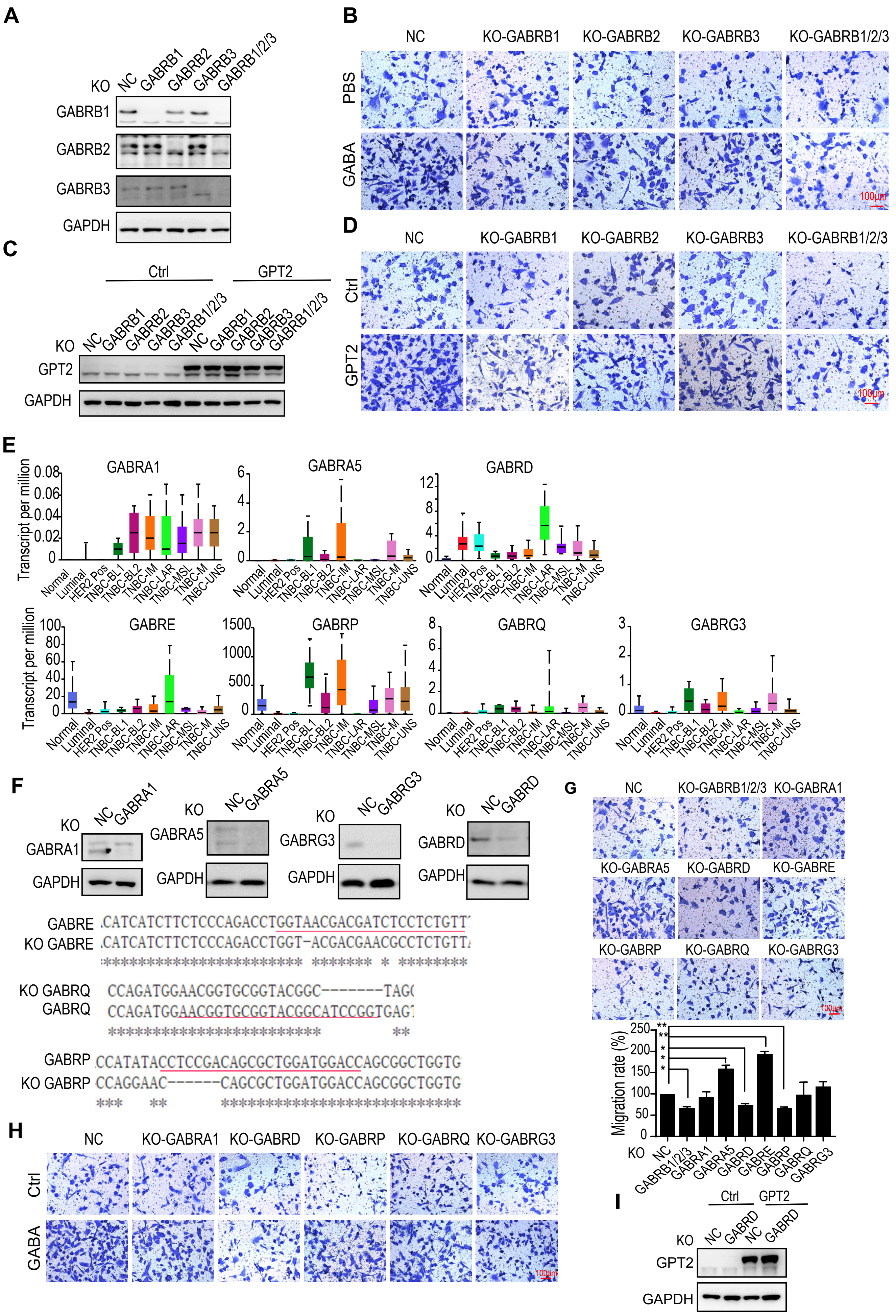

A
KO NC GABRB1 GABRB2 GABRB3 GABRB1/2/3
GABRB1
GABRB2
GABRB3
GAPDH
B
NC KO-GABRB1 KO-GABRB2 KO-GABRB3 KO-GABRB1/2/3
PBS
GABA
100μm
C
Ctrl GPT2
KO NC GABRB1 GABRB2 GABRB3 GABRB1/2/3 NC GABRB1 GABRB2 GABRB3 GABRB1/2/3
GPT2
GAPDH
D
NC KO-GABRB1 KO-GABRB2 KO-GABRB3 KO-GABRB1/2/3
Ctrl
GPT2
100μm
E
Transcript per million
GABRA1
GABRA5
GABRD
GABRE
GABRP
GABRQ
GABRG3
Normal Luminal HER2 Pos TNBC-BL1 TNBC-BL2 TNBC-IM TNBC-LAR TNBC-MSL TNBC-M TNBC-UNS
F
KO NC GABRA1
GABRA1
GAPDH
KO NC GABRA5
GABRA5
GAPDH
KO NC GABRG3
GABRG3
GAPDH
KO NC GABRD
GABRD
GAPDH
GABRE CATCATCTTCTCCCAGACCTGGTAACGACGATCTCCTCTGTT
KO GABRE CATCATCTTCTCCCAGACCTGGT-ACGACGAACGCCTCTGTT
KO GABRQ CCAGATGGAACGGTGCGGTACGGC-------TAG
GABRQ CCAGATGGAACGGTGCGGTACGGCATCCGGTGAG
GABRP CCATATACCTCCGACAGCGCTGGATGGACCAGCGGCTGGTG
KO GABRP CCAGGAAC------CAGCGCTGGATGGACCAGCGGCTGGTG
G
NC KO-GABRB1/2/3 KO-GABRA1
KO-GABRA5 KO-GABRD KO-GABRE
KO-GABRP KO-GABRQ KO-GABRG3
100μm
Migration rate (%)
KO NC GABRB1/2/3 GABRA1 GABRA5 GABRD GABRE GABRP GABRQ GABRG3
H
NC KO-GABRA1 KO-GABRD KO-GABRP KO-GABRQ KO-GABRG3
Ctrl
GABA
100μm
I
Ctrl GPT2
KO NC GABRD NC GABRD
GPT2
GAPDH

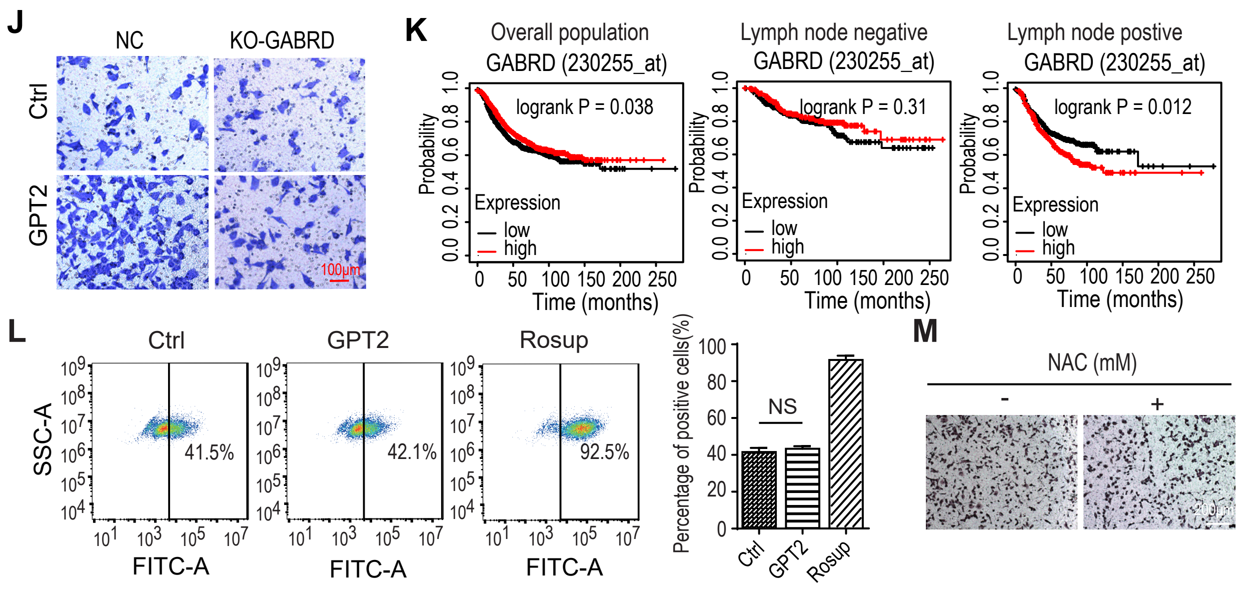

Figure S3 legend

A. GABRB1, GABRB2, GABRB3 and entirely knocked out BT549 stable cells were identified by Western blot.

B. The effects of $GABA_A$ receptor β subunit deletion in BT549 on cell migration and the response to GABA after β subunit deletion were detected by transwell assay. Cells were pretreated with 100 uM GABA for 24h.

C. Overexpression GPT2 in GABRB1, GABRB2, GABRB3 and entirely knocked out BT549 stable cells were identified by Western blot.

D. The effects of $GABA_A$ receptor β subunit deletion in BT549 on GPT2-induced cell migration, detected by transwell assay.

E. Screening high expression GABR genes in TNBC based on TCGA database (http://ualcan.path.uab.edu).

F. GABRA1, GABRA5, GABRD, GABRE, GABRP, GABRQ, and GABRG3 knocked out BT549 stable cells were identified by Western blot or genome sequencing.

G. The effect of GABRA1, GABRA5, GABRD, GABRE, GABRP, GABRQ, or GABRG3 knockout on cell migration was detected by transwell assay.

H. The effect of GABA on cell migration after GABRA1, GABRD, GABRP, GABRQ, or GABRG3 knockout in BT549 cells was detected by transwell assay.

I. Overexpression GPT2 in GABRD knocked out BT549 stable cells were identified by Western blot.

J. The effects of GABRD knockout in BT549 on GPT2-induced cell migration, detected by transwell assay.

K. The expression level of GABRD is negatively correlated with the overall survival rate of patients with node-positive breast cancer (https://kmplot.com/analysis/).

L. Detection of ROS in BT549 cells overexpressing GPT2. NS: $p > 0.05$.

M. The oxidative redutase NAC did not significantly affect breast cancer cell migration. The concentration of NAC was 8 mM.

Figure S4

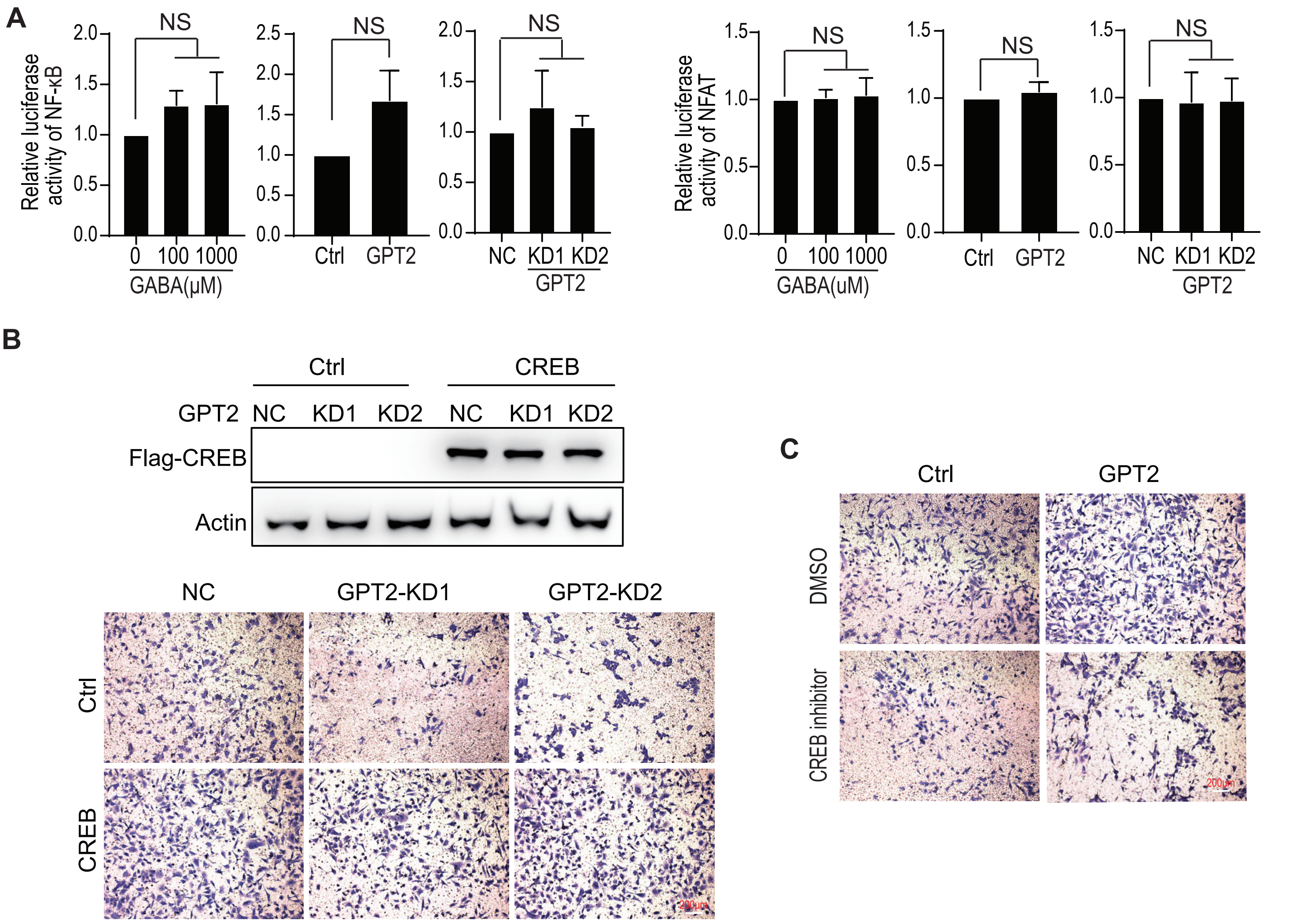

Figure S4 legend:

A. The effect of GPT2/GABA on the activation of NF-κB and NFAT, detected by lucifirase assay in BT549. NS: $p > 0.05$.

B. Overexpression CREB in GPT2 knockdown BT549 stable cells were identified by Western blot，and the effect of CREB overexoression on the GPT2-induced breast cancer migration by transwell assay.

C. The effect of CREB inhibitor 666-15 on the GPT2-induced breast cancer migration by transwell assay. Cells were pretreated by 10 μM 666-15 for 24h.
